# Double-Constraint Inpainting Model of a Single-Depth Image

**DOI:** 10.3390/s20061797

**Published:** 2020-03-24

**Authors:** Wu Jin, Li Zun, Liu Yong

**Affiliations:** 1 School of Information Science and Engineering, Wuhan University of Science and Technology, Wuhan 430081, China; wujin@wust.edu.cn (W.J.); 141001@xxmu.edu.cn (L.Y.); 2 School of Physics and Electronic Engineering, Xinxiang College, Xinxiang 453000, China

**Keywords:** depth image inpainting, variable splitting technique, low-rank constraint, nonlocal self-similarity constraint

## Abstract

In real applications, obtained depth images are incomplete; therefore, depth image inpainting is studied here. A novel model that is characterised by both a low-rank structure and nonlocal self-similarity is proposed. As a double constraint, the low-rank structure and nonlocal self-similarity can fully exploit the features of single-depth images to complete the inpainting task. First, according to the characteristics of pixel values, we divide the image into blocks, and similar block groups and three-dimensional arrangements are then formed. Then, the variable splitting technique is applied to effectively divide the inpainting problem into the sub-problems of the low-rank constraint and nonlocal self-similarity constraint. Finally, different strategies are used to solve different sub-problems, resulting in greater reliability. Experiments show that the proposed algorithm attains state-of-the-art performance.

## 1. Introduction

With the rapid development of RGB-D (red green blue-depth) sensors [1,2,3,4,5,6], such as the Kinect sensor, colour images and depth images can be obtained simultaneously. Depth images are widely used in 3D reconstruction, 3D videos and medical intelligence and are therefore a research area focus for image processing and computer vision. Initially, the development of depth images was limited by the cost-effectiveness of devices used to acquire depth images [7,8,9,10]. In 2010, Microsoft launched the Kinect sensor to acquire depth images, and it attracted wide attention and expanded the associated applications.

In practical applications, depth images are of low quality and have black holes. Black holes represent missing depth information, and the black-hole filling problem is solved via depth image inpainting. At present, depth image inpainting methods can be divided into two categories according to whether the corresponding colour images are guided.

The first method relies on the use of corresponding colour images as a guide. Liu et al. [11] proposed a robust optimisation framework for colour image-guided depth image restoration. This method performs well in suppressing texture artefacts. Lee et al. [12] proposed an adaptive edge-oriented smoothing process based on the characteristics of holes with or without vertical lines in the colour image. The proposed method represents a good trade-off between time savings, hole reduction, and virtual view quality. Lei et al. [13] proposed a credibility-based multi-view depth image fusion strategy to refine images. This method considers the view synthesis quality and inter-view correlation in an improved repair approach.

The second method does not use corresponding colour images as a guide. Shen et al. [14] proposed the inpainting method using a weighted joint bilateral filter and fast marching. This method has obtained the best performance in improving depth images by producing smooth and edge regions. Buyssens et al. [15] proposed a suitable method for recovering the lost structures of objects to in-paint depth images in a geometrically plausible manner. Lu et al. [16] proposed a method of inpainting depth images through similar patches in a matrix and enforced low-rank subspace constraints, thereby attaining good performance. Xue et al. [17] proposed the low-gradient regularisation method, an effective approach that reduces the penalty for gradient 1 while penalizing non-zero gradients to allow for gradual depth changes.

To reduce the complexity of the inpainting problem, we solve depth image inpainting without corresponding colour images and fully exploit the features of depth images to complete the inpainting task of single-depth image inpainting.

Based on previous work, the use of a single-image property as one constraint is not sufficient to obtain satisfying inpainting results. Consequently, we use more than one constraint to perform depth image inpainting.

Depth images can be regarded as textureless natural images that consist of many similar flat areas and few edge areas. Depth images therefore have the characteristics of a low rank and nonlocal self-similarity. Due to its textureless property, the effect of the low-rank constraint on inpainting will be too great and false details will be created. Therefore, we introduce the nonlocal self-similarity constraint. First, we regard the depth image as a matrix, and the corresponding low-rank reconstruction model is built based on the low-rank structure of the matrix in the image. We then introduce the nonlocal self-similarity constraint to improve the depth image results. The contributions of this paper are summarised as follows.

1. Rather than the traditional single-constraint method, we adopt a double-constraint method. According to the characteristics of the depth image, we combine the low-rank constraint and nonlocal self-similarity constraint.
2. We adopt the split Bregman algorithm, which is a variable splitting technique, to divide depth image inpainting into sub-problems, thus reducing the complexity of the solution.
3. We use different strategies to solve depth image inpainting: weighted Schatten *p*-norm minimisation as the low-rank constraint and nonlocal statistical modelling as the nonlocal self-similarity constraint. The proposed method achieves better performance.

The remainder of our paper is organised as follows. In Section 2, we present the related work. In Section 3, we describe the details of the depth image inpainting method based on the double-constraint. In Section 4, we present the experimental results. In Section 5, we summarise the paper.

## 2. Related Work

### 2.1. Depth Images

**Figure 1:**
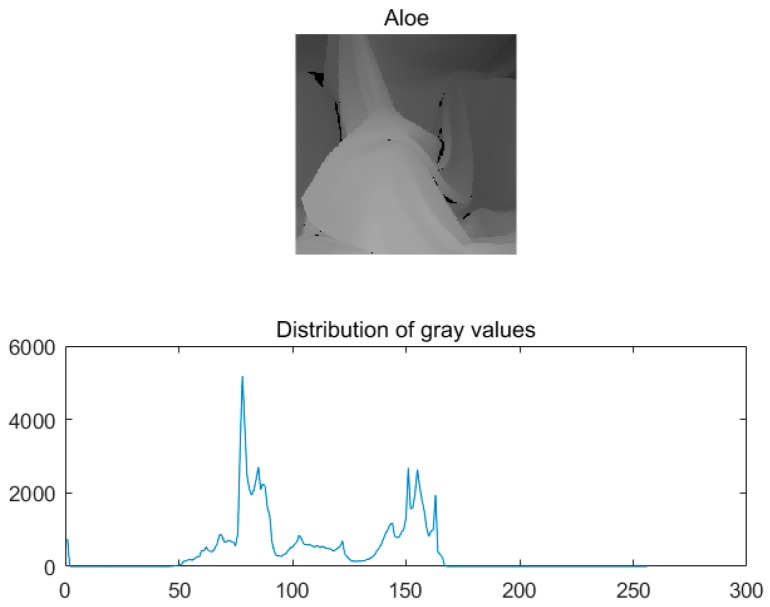
Distribution of grey values.

Depth images are greyscale images with pixel values of 0–255, and the greyscale value of the pixel represents the distance between the spatial scene and the camera. In general, the closer the area is to the camera, the greater the depth, while the farther away the area is to the camera, the smaller the depth. The depth image consists of the most similar flat regions and a few edge regions which contain a large number of regions with the same grey value. A continuous distribution with the same depth values exists inside the object, and the gradient is 0. Depth value mutations and gradients are observed at the edges. The Aloe depth image [18,19,20] is taken as an example, and the grey value of the depth image is as shown in Figure 1.

As shown in Figure 1, the distribution of grey values in the depth image is very concentrated. Depth images have the characteristics of a low rank and nonlocal self-similarity.

### 2.2. Low-Rank Constraint and Nonlocal Self-Similarity Constraint

The single-depth image inpainting problem is transformed into the following mathematical expression:

(1) $$x=\arg\min_{x}\frac{1}{2}||Hx-y|{|}_{2}^{2}+\lambda \cdot \psi (x).$$

where x is the intact depth image; y is the degraded depth image; $||Hx-y|{|}_{2}^{2}$ is the data-fidelity term; ψ(x) is the regularisation term; λ is the weight parameter; and H is a binary template. We attempt to obtain a potential depth image x from the degraded depth image y. According to the characteristics of the depth image, we combine the low-rank constraint and nonlocal self-similarity constraint. Equation (1) can be converted into Equation (2):

(2) $$x=\arg\min_{x}\frac{1}{2}||Hx-y|{|}_{2}^{2}+\lambda_{1}\cdot \Psi_{\mathrm{LR}}(x)+\lambda_{2}\cdot \Psi_{\mathrm{NSS}}(x).$$

where $\Psi_{\mathrm{LR}}(x)$ represents the low-rank regularisation term, $\Psi_{\mathrm{NSS}}(x)$ represents the nonlocal self-similarity regularisation term, and $\lambda_{1}$ and $\lambda_{2}$ are the weight parameters.

At present, the solution methods for low-rank matrices can be divided into two categories: low-rank matrix decomposition and rank minimisation. The commonly used low-rank matrix decomposition method mostly adopts the singular decomposition technique, which uses the *f*-norm fidelity loss to trim the singular value matrix to obtain the optimal rank approximation solution. The rank minimisation method mainly uses the relaxation method to minimise the rank and estimate the lowest rank for reconstruction. The latter method has a better recovery performance. Therefore, we use the nuclear norm minimisation-based (NNM-based) method for depth image inpainting. 

Scholars have conducted some research into the solution of the NNM problem. In [21], under certain conditions, the NNM method is used to achieve reconstruction with limited information. In [22], the soft threshold operation is applied to the NNM method for matrix filling purposes in a very small storage space. In [23], low-level visual problems are solved by minimizing the sum of partial singular values. In [24], weighted nuclear model minimisation is proposed, and the method adaptively weights singular values differently, which improves the applicability and flexibility of low-quality images.

Compared with NNM, weighted Schatten *p*-norm minimisation (WSNM) [25] can better approximate the original low-order hypothesis and consider the importance of different components. WSNM can be effectively applied to obtain the global optimal solution. Therefore, we use WSNM as the low-rank constraint.

In addition to the low-rank constraint, nonlocal self-similarity is another important feature of depth images. This feature can describe the structure repetition characteristic of the nonlocal area of the depth image and preserve the edge and detail effectively.

The repeatability of the nonlocal self-similarity description advanced mode has enabled remarkable achievements in the field of image reconstruction. Buades et al. [26] proposed an effective denoising model called nonlocal means (NLMs) via the degree of similarity among surrounding pixels for denoising tasks. Jung et al. [27] proposed a class of restoration algorithms for colour images based upon the Mumford–Shah model and nonlocal image information. These algorithms are defined to work in a small local neighbourhood and are sufficient to denoise smooth regions with sharp boundaries. In [28], a nonlocal self-similarity constraint is introduced into the overall cost functional to improve the robustness of the model. The proposed method outperforms many existing image reconstruction methods. The nonlocal self-similarity constraint produces superior results with sharper image edges.

However, traditional nonlocal self-similarity constraints fail to recover accurate structures in depth images. We use the nonlocal self-similarity (NLSM) [29] of the three-dimensional transformation domain as the constraint term. Compared with traditional methods, the NLSM of the three-dimensional transformation domain represents self-similarity more effectively and has adaptive performance.

## 3. Double-Constraint Model 

### 3.1. Similar Block Group and NLSM Model

**Figure 2:**
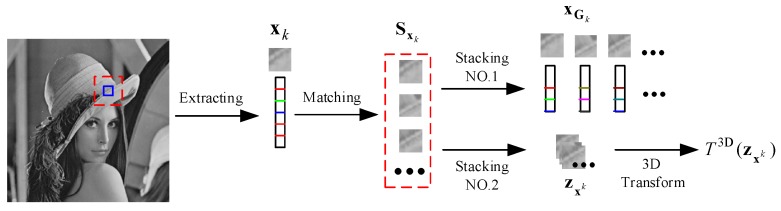
Construction of the similar block group and the non-local self-similar statistical model.

The similar block group and NLSM are based on similar blocks. The construction is shown in Figure 2.

As shown in Figure 2, we first divide the image x into pixel blocks of size $\sqrt{B_{s}}\times \sqrt{B_{s}}$, and each pixel block is expressed in vector form $x_{k}$, where *k* = 1, 2, 3, …, *n*. Then, for each patch $x_{k}$ denoted by a blue mark, in the red window, we determine its c similar patches, which compose set $S_{x_{k}}$.

In the first stacking, all patches in set $S_{x_{k}}$ are arranged in a matrix to obtain similar groups, with $x_{G_{k}}\in R^{B_{s}\times c}$. Due to the characteristics of the greyscale values in the depth image, the principle of similarity matching is the SSD principle.

In the second stacking, all patches in set $S_{x_{k}}$ are stacked in a three-dimensional $z_{x_{k}}$. By orthogonal three-dimensional transformation $T^{3D}$, the coefficients of three-dimensional arrangement $T^{3D}(z_{x_{k}})$ are obtained.

### 3.2. Solution of Depth Image Inpainting

By introducing variables u and v, we can transform Equation (2) into an equivalent constrained form as follows:

(3) $$x=\arg\min_{x}\frac{1}{2}||Hx-y|{|}_{2}^{2}+\lambda_{1}\cdot \Psi_{\mathrm{LR}}(u)+\lambda_{2}\cdot \Psi_{\mathrm{NSS}}(v)\begin{array}{cc} & s.t \end{array}\begin{array}{cc} & \left[\begin{array}{c} u\\ v \end{array}\right] \end{array}=Gx$$

where $G={\left[I,I\right]}^{T}$.

By introducing externalised constraints, we arrive at the following:

(4) $$x=\arg\min_{x}\frac{1}{2}||Hx-y|{|}_{2}^{2}+\lambda_{1}\cdot \left|\right|u{\left|\right|}_{w,s_{p}}^{p}+\lambda_{2}\cdot \left|\right|\Theta_{V}{\left|\right|}_{1}.$$

where $\left|\right|u{\left|\right|}_{w,s_{p}}^{p}=\sum_{i=1}^{\min\{n,m\}}\omega_{i}\sigma_{i}^{p}=tr(W\Delta^{p})$, $\left|\right|\Theta_{V}{\left|\right|}_{1}={\sum_{i=1}^{n}\left||T^{3D}(z_{x^{k}})|\right|}_{1}$

Then, we use the split Bregman algorithm [30] to transform complex problems into sub-problems that are easy to solve:

(5) $$x^{t+1}=\arg\min_{x}\frac{1}{2}||Hx-y|{|}_{2}^{2}+\frac{\mu }{2}||x-u^{t}-b^{t}|{|}_{2}^{2}+\frac{\mu }{2}\left|\right|x-v^{t}-b^{t}{\left|\right|}_{2}^{2}$$

(6) $$u^{t+1}={\mathrm{argmin}}_{u}\lambda_{1}\cdot \left|\right|u{\left|\right|}_{w,s_{p}}^{p}+\frac{\mu }{2}||x^{t+1}-u^{t}-b^{t}|{|}_{2}^{2}$$

(7) $$v^{t+1}={\mathrm{argmin}}_{v}\lambda_{2}\cdot \left|\right|\Theta_{v}{\left|\right|}_{1}+\frac{\mu }{2}||x^{t+1}-v^{t}-c^{t}|{|}_{2}^{2}$$

where $b^{t+1}=b^{t}-(x^{t+1}-u^{t+1})$ and $c^{t+1}=c^{t}-(x^{t+1}-v^{t+1})$.

Other variable splitting techniques, such as the half quadratic splitting method, can also be used to transform complex problems into sub-problems.

For conciseness and to avoid ambiguity, the iterations are omitted in the following discussion of the sub-problems.

#### 3.2.1. Sub-Problem x

The split Bregman algorithm converts Equation (4) into three sub-problems. Equation (5) represents the minimisation problem of sub-problem x transformed into a strict convex quadratic function. The closed solution of Equation (5) can be obtained as follows:

(8) $$x={\left(H^{T}H+2\mu I\right)}^{-1}[H^{T}y+\mu (u+v+b+c)]$$

#### 3.2.2. Sub-Problem u

According to the solution of u, Equation (6) can be converted into the following equation:

(9) $$u={\mathrm{argmin}}_{u}||r_{u}-u|{|}_{2}^{2}+\frac{2\lambda_{1}}{\mu }\left|\right|u{\left|\right|}_{w,s_{p}}^{p}$$

where $r_{u}=x-b$. Let $e_{u}=r_{u}-u$, which represents the residual. Taking the Aloe depth image as an example, we select deblurring replacement inpainting to carry out the simulation experiments. The reasons why we chose image deblurring as an example for verification are twofold: 1. we have an accurate original depth image for objective data comparison; and 2. image deblurring and inpainting both satisfy Equation (1).

We chose to approximate the Aloe depth image as u. Then, the residual distribution of the kth iteration can be obtained.

**Figure 3:**
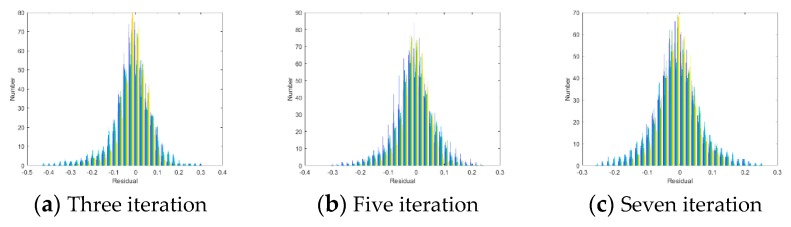
Distribution of $e_{u}$ for different iterations.

We first performed 3×3 uniform blur kernel operations on the Aloe depth image and then added Gaussian noise with a standard deviation of 1 to obtain a blurred depth image. Figure 3 shows the distribution of the residuals in the experiments with three, five and seven iterations.

As shown in Figure 3, in each iteration, the distribution of $e_{u}$ is suitably characterised by a generalised Gaussian distribution with a zero mean.

According to the experiments, we formulate the following hypothesis [24,31,32]: in each iteration, the residuals satisfy a generalised Gaussian distribution with a zero-mean. In each iteration, the following equation is satisfied:

(10) $$\frac{1}{N}\left|\right|r_{u}-u{\left|\right|}_{2}^{2}=\frac{1}{K}\sum_{k=1}^{n}\left|\right|r_{G_{k}}-u_{G_{k}}|{|}_{F}^{2}$$

By substituting Equation (10) into Equation (9), we can obtain the following:

(11) $$u={\mathrm{argmin}}_{u_{}}\sum_{k=1}^{n}\frac{\mu N}{2\lambda_{1}K}||r_{G_{k}}-u_{G_{k}}|{|}_{F}^{2}+\left|\right|u_{G_{k}}{\left|\right|}_{w,s_{p}}^{p}$$

For each similar group, we assume that the singular decomposition of $r_{G_{k}}$ is $r_{G_{k}}=U\Sigma V^{T}$, with $\Sigma =\mathrm{diag}(\sigma_{1},\ldots ,\sigma_{r})$, which is non-ascending. According to Von Neumann’s trace inequality theorem [33], the solution of Equation (9) transforms the solution of $\Delta =\mathrm{diag}(\delta_{1},\ldots ,\delta_{r})$ into $u_{G_{k}}=Q\Delta R^{T}$. The solution equation is as follows.

(12) $$\{\begin{array}{c} \underset{\delta_{1}\ldots \delta_{r}}{\min}\sum_{i=1}^{r}[{\left(\delta_{i}-\sigma_{i}\right)}^{2}+\omega_{i}\delta_{i}^{p}],i=1,\ldots r\\ s.t\begin{array}{cc} & \delta_{i}\geq 0\begin{array}{ccc} & and & \delta_{i}\geq \delta_{j} \end{array},\begin{array}{cc} & for\begin{array}{cc} & i\leq j \end{array} \end{array} \end{array} \end{array}$$

The solution of Equation (11) can be converted into the following equation:

(13) $$\underset{\delta_{i}\geq 0}{\min}f_{i}(\delta )={\left(\delta_{i}-\sigma_{i}\right)}^{2}+\omega_{i}\delta_{i}^{p},i=1,\ldots ,r$$

Equation (13) can be solved by using the generalised soft threshold (GST) algorithm [34]. 

If p and $\omega_{i}$ are determined, according to the GST algorithm, there is a special threshold $\sigma_{i}\geq \tau_{p}^{GST}(\omega_{i})$ that satisfies the following equation:

(14) $$\tau_{p}^{GST}(\omega_{i})={\left(2\omega_{i}(1-p)\right)}^{\frac{1}{2-p}}+\omega_{i}p{\left(2\omega_{i}(1-p)\right)}^{\frac{p-1}{2-p}}$$

If $\sigma_{i}<\tau_{p}^{GST}(\omega_{i})$, then the following holds:

(15) $$f_{i}(\delta )=\sigma_{i}{}^{2}+\omega_{i}\delta_{i}^{p},i=1,\ldots ,r$$

That is, $\delta_{i}=0$, which is the global minimum.

If $\sigma_{i}\geq \tau_{p}^{GST}(\omega_{i})$, then $f_{i}(\delta )$ has the minimum value of $S_{p}^{GST}(\sigma_{i};\omega_{i})$, which can be obtained by solving the following equation:

(16) $$S_{p}^{GST}(\sigma_{i};\omega_{i})-\sigma_{i}+\omega_{i}p{\left(S_{p}^{GST}(\sigma_{i};\omega_{i})\right)}^{p-1}=0$$

Then, sub-problem u is solved.

#### 3.2.3. Sub-Problem v

According to the solution of v, Equation (7) can be converted into the following equation:

(17) $$v={\mathrm{argmin}}_{v}\frac{1}{2}||r_{v}-v|{|}_{2}^{2}+\frac{2\lambda_{2}}{\mu }\left|\right|\Theta_{v}{\left|\right|}_{1}$$

where $r_{v}=x-c$. Let $e_{v}=r_{v}-v$, which represents the residual. $e_{v}$ and $e_{u}$ have the same property. As a result, Equation (17) can be converted into Equation (18):

(18) $$v={\mathrm{argmin}}_{v}\frac{1}{2}||\Theta_{r_{v}}-\Theta_{v}|{|}_{2}^{2}+\frac{2K\lambda_{2}}{N\mu }\left|\right|\Theta_{v}{\left|\right|}_{1}$$

In the above equation, any elements of $\Theta_{v}$ can be solved separately; therefore, we use the soft threshold [35]. to solve Equation (18).

(19) $$\Theta_{v}=\mathrm{soft}(\Theta_{r_{v}},\frac{2K\lambda_{2}}{N\mu })$$

Namely,

(20) $$\begin{array}{ll} \Theta_{v}(j) & =\mathrm{sgn}((\Theta_{x}(j))\max\{|\Theta_{r_{v}}(j)-\frac{2K\lambda_{2}}{N\mu }|,0\}\\ & =\{\begin{array}{cc} \Theta_{r_{V}}(j)-\frac{2K\lambda_{2}}{N\mu },\; & \Theta_{r_{v}}(j)\in (\frac{2K\lambda_{2}}{N\mu },+\infty )\\ 0,\; & \Theta_{r_{v}}(j)\in [-\frac{2K\lambda_{2}}{N\mu },\frac{2K\lambda_{2}}{N\mu }]\\ \Theta_{r_{v}}(j)+\frac{2K\lambda_{2}}{N\mu },\; & \Theta_{r_{v}}(j)\in (-\infty ,-\frac{2K\lambda_{2}}{N\mu }) \end{array} \end{array}$$

In summary, all the sub-problems in our proposed algorithm are solved. The flow chart of the algorithm is shown in Table 1.

## 4. Experiments

### 4.1. Depth Image Inpainting

In this paper, the hardware simulation platform was supported by a Lenovo R720 computer (Lenovo, Beijing, China) and the software simulation platform was MATLAB R2017a (The MathWorks, Inc., Massachusetts, USA).

The inpainting effect as analysed by subjective visual requirements and objective parameters. The objective metrics could be assessed on the basis of two objective metrics: the peak signal-to-noise ratio (PSNR) [36] and the feature similarity (FSIM) [37]. PSNR uses the ratio of the maximum semaphore to noise intensity to measure image quality, which is easy to calculate and understand and can reflect the image quality. FSIM is a novel low-level feature similarity parameter. Phase congruency is a dimensionless measure of the significance of a local structure, and it is used as the primary feature in FSIM.

We used a dataset that included the Middleburry datasets [20,21,22] and NYU v2 dataset [38]. For the comparison algorithm, we proposed similar algorithms, namely NNM [8] and WSNM [18].

**Figure 4:**
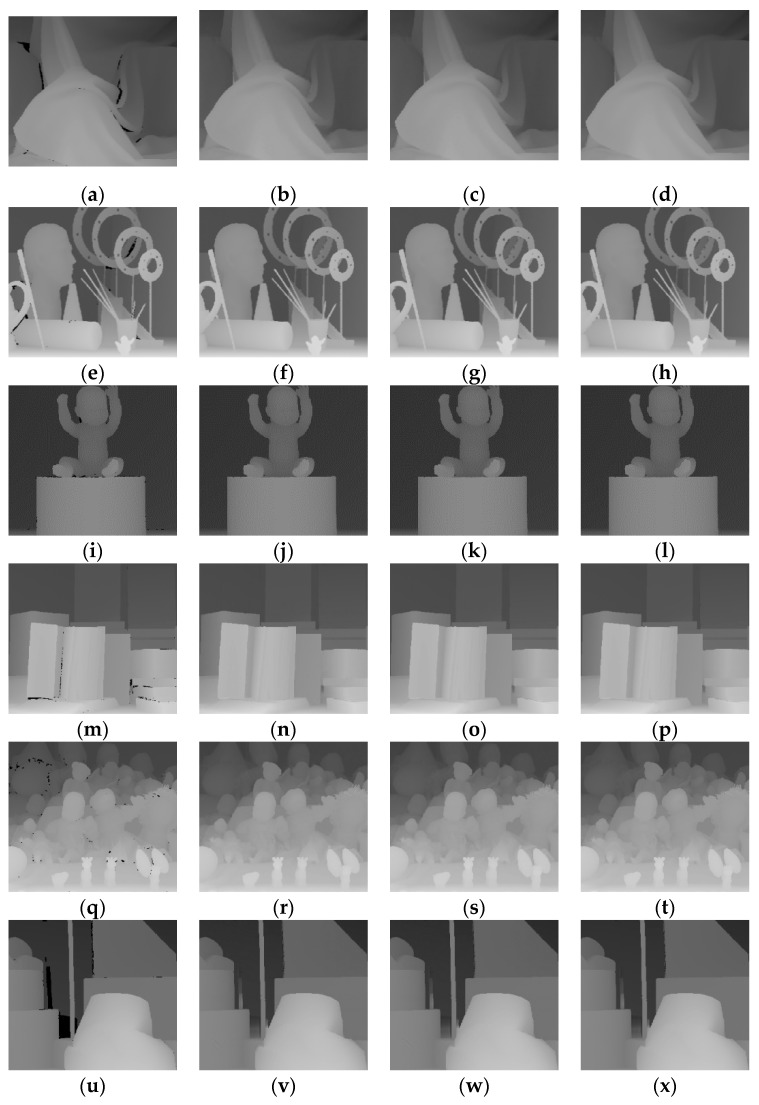
Visual quality comparison of the inpainting result (1): (**a**) Aloe depth image; (**b**–**d**) inpainting effects of the nuclear norm minimisation (NNM) algorithm, weighted Schatten *p*-norm minimisation (WSNM) algorithm and proposed algorithm for the Aloe depth image; (**e**) Art depth image; (**f**–**h**) inpainting effects of the NNM algorithm, WSNM algorithm and the proposed algorithm for the Art depth image; (**i**) Baby depth image; (**j**–**l**) inpainting effects of the (nuclear norm minimisation) NNM algorithm, WSNM algorithm and the proposed algorithm for the Baby depth image; (**m**) Books depth image; (**n**–**p**) inpainting effects of the NNM algorithm, WSNM algorithm and the proposed algorithm for the Books depth image; (**q**) Dolls depth image; (**r**–**t**) inpainting effects of the NNM algorithm, WSNM algorithm and the proposed algorithm for the Dolls depth image; (**u**) Lam depth image; and (**v**–**x**) inpainting effects of the NNM algorithm, WSNM algorithm and the proposed algorithm for the Lam depth image.

In experiment 1, the depth images were obtained directly from the Middleburry datasets. The area requiring repair was the actual situation. as shown in Figure 4.

As shown in Figure 4, all three algorithms met the visual requirements and no obvious repair marks occurred. However, the details varied from image to image. The NNM algorithm and WSNM algorithm both resulted in the smoothing of boundaries. Our proposed algorithm could reduce this situation, as shown in the enlarged portion of the figure in the red box.

As summarized in Table 2, the proposed algorithm was superior to the other two algorithms and the objective data were improved.

**Figure 5:**
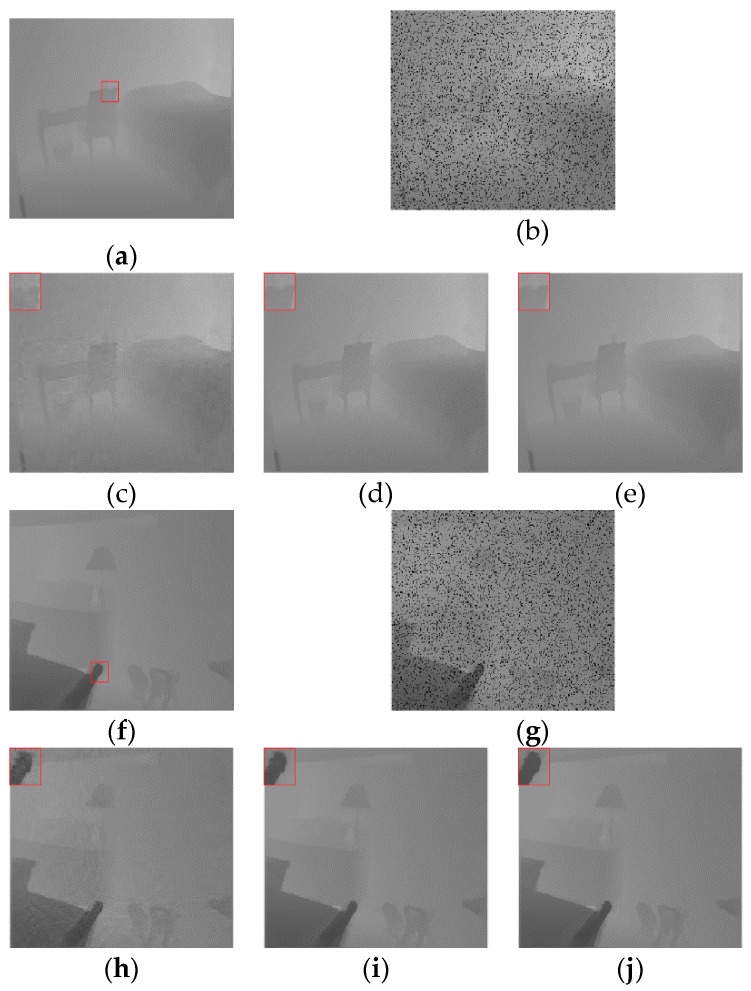

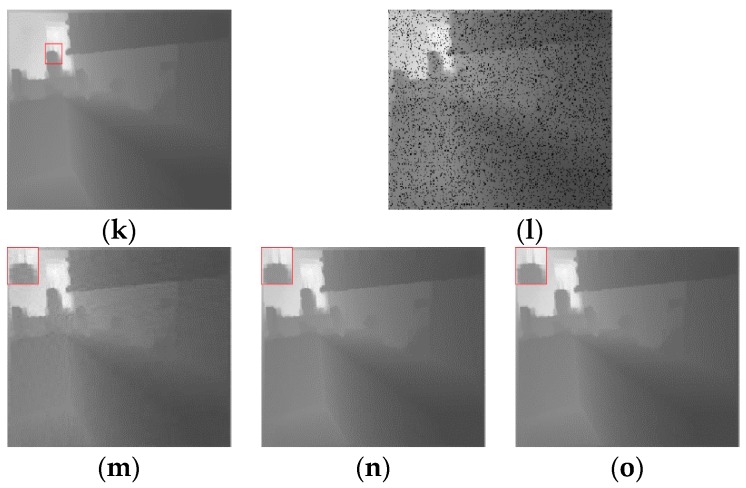
Visual quality comparison of the inpainting result (2): (**a**) Bedroom depth image; (**b**) Corrupted bedroom depth image with 10% pixels missing; (**c**–**e**) Inpainting effects of the NNM algorithm, WSNM algorithm and the proposed algorithm; (**f**) Lamp depth image; (**g**) Corrupted lamp depth image with 10% pixels missing; (**h**–**j**) Inpainting inpainting effects of the NNM algorithm, WSNM algorithm and the proposed algorithm; (**k**) Kitchen depth image; (**l**) Corrupted kitchen depth image with 10% pixels missing; (**m**–**o**) Inpainting effects of the NNM algorithm, WSNM algorithm and the proposed algorithm.

In experiment 2, the depth images were obtained directly from the NYU v2 dataset. The area requiring repair was the 10% data loss area, as shown in Figure 5.

Subjectively, as shown in Figure 5, the NNM algorithms were not able to meet the visual requirements, and the image was blurred. The WSNM algorithm and our algorithm both met the visual requirements. However, the edge processing of algorithm WSNM was not good. Our proposed algorithm could reduce this situation, as shown in the enlarged portion of the figure in the red box.

Objectively, as summarized in Table 3, the proposed algorithm was superior to the other two algorithms. All the objective data were improved.

In summary, the proposed algorithm has certain advantages and can be used in depth image inpainting applications.

### 4.2. Parameter Influence

To discuss the influence of different parameters on the proposed algorithm, we analysed the Aloe, Art and Books depth images.

#### 4.2.1. Number of Best-Matched Patches

**Figure 6:**
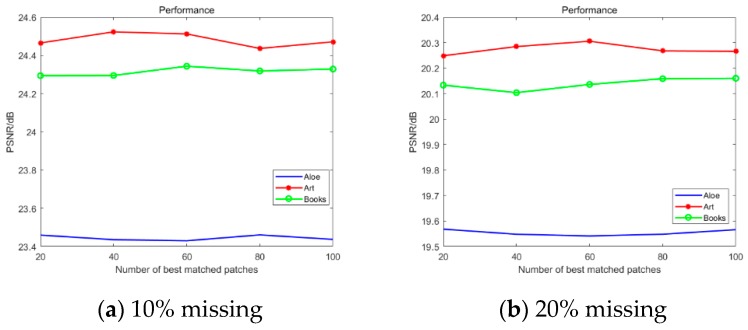
Performance comparison with best-matched patches.

The manual damage in this section was 10% data loss and 20% data loss. As shown in Figure 6, when were number of pixel blocks of a similar group was in the range of 20–100, the experimental curves are relatively flat. That is, the proposed algorithm was insensitive to the number of pixel blocks. Therefore, the number of pixel blocks was 60.

#### 4.2.2. Algorithm Stability

**Figure 7:**
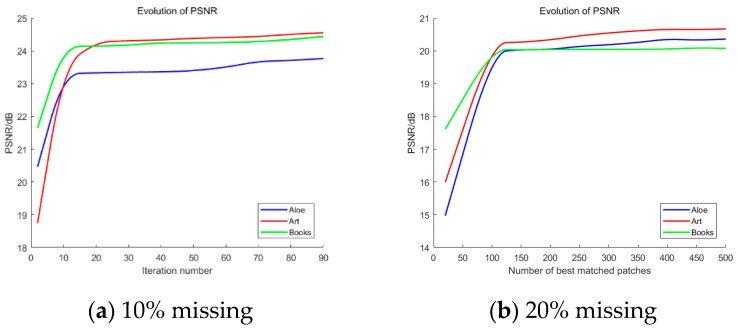
Stability of the proposed algorithm.

The manual damage in this section was 10% data loss and 20% data loss. Since the objective function is non-convex, it was difficult to mathematically prove the global convergence of the proposed algorithm. We obtained experimental data and empirically verified the stability of the proposed algorithm. As shown in Figure 7, the number of iterations increased, the PSNR increased monotonically and eventually stabilised. Therefore, the stability of the proposed algorithm could be verified.

#### 4.2.3. Influence of p

**Figure 8:**
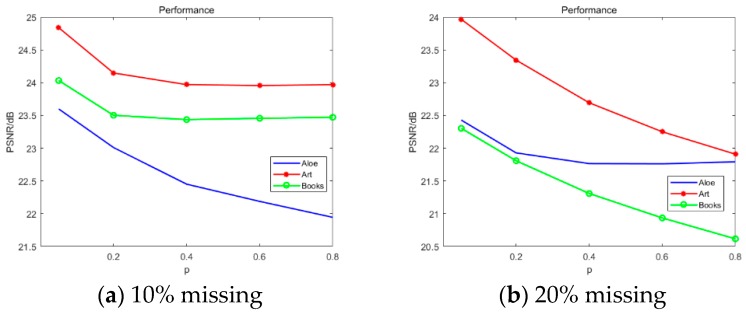
Performance comparison with p.

**Figure 9:**
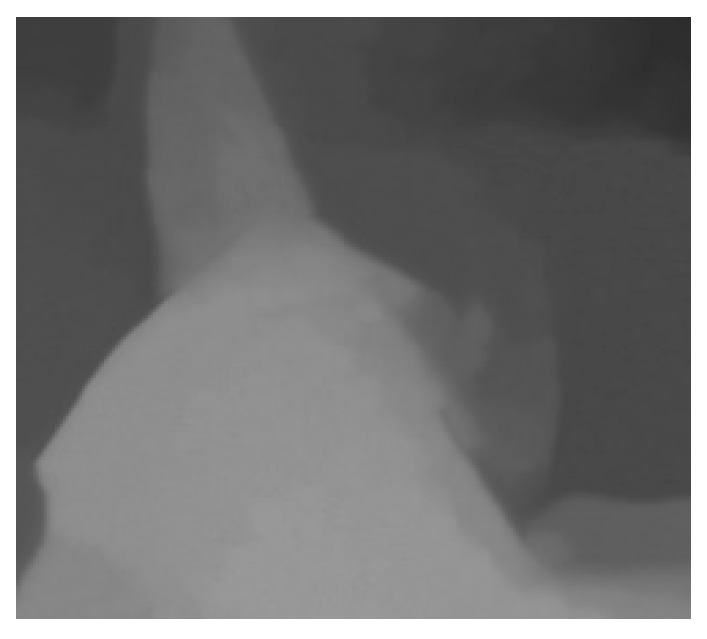
Visual quality.

The manual damage in this section was 10% and 20% data loss. Figure 8 shows that the value of p was small and the objective data were improved. However, p was too small and excessive smoothing occurred. As shown in Figure 9, p was 0.05. Therefore, we chose p=0.2, which agreed with [39].

## 5. Conclusions

The main research topic in our paper is depth image inpainting. The proposed method is based on the fact that depth in-painted images using a low-rank structure and nonlocal self-similarity can fully exploit the features of depth images to complete the inpainting task. The experiments prove that, regardless of the subjective visual effect and objective contrast data, the proposed algorithm can obtain a better repair effect and has a certain practical application value.

## Figures and Tables

**Table 1:** Complete description of the proposed method.

**Input:** The observed depth image y, the degraded operator H
**Output:** The restored depth image x
**Repeat**
**Step 1:** Update **x** by Equation (8)
**Step 2:** For each group $u_{G_{k}}$
**(1)** The singular value decomposition of $r_{G_{k}}$
**(2)** Update $u_{G_{k}}$ by Equation (12)
Aggregate $u_{G_{k}}$ to form u
**Step 3:** Update **v** by Equation (20)
**Until maximum iteration number is reached**

**Table 2:** Peak signal-to-noise ratio (PSNR)/feature similarity (FSIM) in experiment (1).

Image	Algorithm (PSNR/FSIM)		
	NNM	WSNM	Proposed
Aloe	26.0395/0.9571	26.0767/0.9628	**26.1296/0.9705**
Art	26.8853/0.9366	27.1790/0.9825	**27.1833/0.9835**
Baby	30.0559/0.9413	30.2569/0.9902	**30.3200/0.9932**
Books	27.4590/0.9674	28.1774/0.9632	**28.1806/0.9752**
Dolls	29.2717/0.9758	29.0181/0.9739	**29.1254/0.9745**
Lam	23.5473/0.9756	24.4459/0.9761	**24.4534/0.9761**

**Table 3:** PSNR/FSIM in experiment (2).

Image	Algorithm (PSNR/FSIM)		
	NNM	WSNM	Proposed
Bedroom	23.0866/0.9475	23.4500/0.9798	**23.5326/0.9820**
Lamp	23.9880/0.8252	24.1906/0.8594	**24.2457/0.8823**
Kitchen	24.7820/0.8763	26.3996/0.8822	**26.5009/0.9029**
